# Biocompatibility of vertically aligned multi-walled carbon nanotube scaffolds for human breast cancer cell line MDA-MB-231

**DOI:** 10.1007/s40204-017-0078-6

**Published:** 2017-11-16

**Authors:** E. M. Akinoglu, K. Ozbilgin, P. Kilicaslan Sonmez, M. M. Ozkut, M. Giersig, S. Inan, E. Gumustepe, C. Kurtman

**Affiliations:** 1 International Academy of Optoelectronics at Zhaoqing, South China Normal University, Zhaoqing, Guangdong China; 2 Department of Physics, Freie Universität Berlin, Berlin, Germany; 3 Department of Histology and Embryology, Faculty of Medicine, Celal Bayar University, Uncubozköy, Manisa, Turkey; 4 Department of Histology and Embryology, Faculty of Medicine, Near East University, Manisa, Turkey; 5 Department of Histology and Embryology, Faculty of Medicine, Izmir University of Economy, Izmir, Turkey; 6 Department of Radiation Oncology, Faculty of Medicine, Ankara University, Ankara, Turkey

**Keywords:** MDA-MB-231, Breast cancer, MWCNTs, Biocompatibility, Immunohistochemistry

## Abstract

The aim of the current study was to determine whether the MWCNT-based scaffold has a suitable structure for cell growth and provides a biocompatible environment for human MDA-MB-231 cell lines. MWCNT-based nanostructured scaffolds were produced by plasma-enhanced chemical vapor deposition (PECVD) technique. MDA-MB-231 cells were seeded on MWCNTs-textured silicon scaffolds and on pristine silicon surfaces. After 1 week of culturing, the scaffolds were prepared for SEM analysis and immunocytochemical staining was performed for the two groups (MWCNT scaffold and pristine silicon surface), using MMP-2, MMP-9, PI3K, AKT and NF-κB primary antibodies. SEM analyses showed that the MDA-MB-231 cells better adhered to the MWCNT-based nanostructured scaffold than the pristine silicon surface. Immunohistochemical activity of the MDA-MB-231 cells on both materials has similar staining with anti-AKT MMP-2, MMP-9 and NF-κB primary antibodies. Therefore, the results of the present study suggest that the MWCNT-based scaffolds enhanced cell adhesion to the scaffold and exhibited more biomimetic properties and physiological adaptation with the potential to be used for in vitro metastasis studies of BrCa cell lines.

## Introduction

Breast cancer (BrCa) is known to be the second most common cause of cancer-related deaths in women. The main mortality rate is very high because of the distant metastasis especially in bones (Torre et al. 2015; Lipton et al. 2009). The interaction between metastatic BrCa cells and the metastatic tissue is critical for the development and progression of metastases. In vitro models and in vivo animal models have been used to investigate tumor progression. For such studies, a scaffold is indispensable to maintain the cell:matrix interactions to enhance cell growth and differentiation of the cells. Two-dimensional (2D) models (in vitro and in vivo) have inherent limitations with regard to controllability, reproducibility and flexibility of design (Khanna and Hunter 2005). However, the investigation of three-dimensional (3D) in vitro biomimetic models can potentially provide a promising tool for future BrCa cell metastasis studies. As these interactions take place at a nanoscale level, it is appropriate and promising to use nanotechnology to design and produce novel scaffolds. Carbon nanomaterials such as graphene, nanotubes and fullerenes are of great interest for these scaffold designs, as carbon is an abundant element and carbon-based nanomaterials are easy to use in their production. Within the former, multi-walled carbon nanotubes (MWCNTs) are not only mechanically strong, chemically robust, inert in various conditions and electrically conductive, but can also be shaped into 3D scaffold structures (Correa-Duarte et al. 2004; Firkowska et al. 2006). A Young’s modulus study showed that assembled polyallylamine hydrochloride/MWCNT composites are significantly softer than layer-by-layer (LBL) assembled films which are based on polyelectrolytes (PAH/poly (acrylic acid) (Pavoor et al. 2004). An extraordinarily high tensile strength of MWCNT-based LBL assembled composites, comparable to ceramics, was reported by Olek et al. (2004). Therefore, scaffolds based on MWCNTs were previously used for culturing of the bone and cartilage cells (Firkowska et al. 2008). In this work, it was aimed to exploit a novel 3D scaffold based on a multi-walled carbon nanotube textured surface for BrCa cell progression studies.

In this context, it is known that the phosphatidylinositol 3-kinase (PI3K/AKT)/mammalian is a target of the rapamycin (mTOR) pathway, which regulates several signaling pathways like cell survival control, apoptosis, proliferation, motility, and adhesion (Xie et al. 2016). Additionally, it is also known that it is frequently activated in numerous human cancers including breast cancer. PI3K signaling is known to be affected at different biological levels such as in amplification, overexpression, translocation, or mutation (Cizkova et al. 2010). Therefore, this pathway plays a pivotal role in the development and maintenance of cancer and may contribute to therapeutic resistance (Baselga 2011).

The nuclear factor-κB (NF-κB) family is a transcription factor and has an important role in the regulation of several genes. In many types of cancer, NF-κB is constitutively activated and contributes critically to cell proliferation, cell adhesion, inflammation, differentiation, angiogenesis, and cancer progression (Hoesel and Schmid 2013). Furthermore, NF-κB regulates the expression of genes involved in anti-apoptosis (Bcl-2 and Bcl-xL), proliferation (COX-2 and cyclin D1), and metastasis (MMP-9). Consequently, NF-κB is involved in many important cellular processes; therefore, investigation of its interaction with other complementary survival pathways like PI3K/Akt is very appealing (Shin et al. 2016).

Cell invasion and metastasis are additionally influenced by matrix metalloproteinases (MMPs) that belong to a zinc-dependent endopeptidase family which essentially degrade the extracellular matrix (ECM). MMP-2 and MMP-9 are the most important proteinases, which can degrade collagen types I/IV, fibronectin, entactin, and elastin. Upregulation of MMP-2 and MMP-9 is very closely related to poor prognosis in breast cancer patients (Li et al. 2004). The expression of MMP-2/9 also depends on the activity of activator protein-1 (AP-1) and NF-κB, as they are the downstream factors of the MAPK signaling pathway.

In this study, it was investigated to see whether a 3D nanoscale MWCNT-based scaffold could be used for tumor progression (including proliferation, migration, and invasion) in human BrCa cell (MDA-MB-231) lines. In this study, electron microscopy and immunohistochemical techniques with PI3K, Akt, NF-κB, MMP-2, and MMP-9 primary antibodies were used to show that MWCNT-textured surfaces enhance cell adhesion without changing the molecular characteristics of the cells.

## Material method

### Scaffold synthesis

Plasma-enhanced chemical vapor deposition (PECVD) was employed to synthesize MWCNT-based nanostructured scaffolds. This technique enabled the growth of vertically aligned MWCNTs on flat silicon surfaces as described before (Trzeciak et al. 2016). Briefly, MWCNTs were grown through the vapor–liquid–solid method (VLS) employing nanosized liquid nickel droplets which act as a catalyst in solid MWCNT formation from gaseous carbon species of decomposed acetylene (C_2_H_2_) gas admixed with ammonia (NH_3_). The growth process resulted in vertically aligned MWCNTs on a silicon surface with the nickel catalysts encapsulated at their tips resembling a nanoscopic bed of nails. The MWCNTs in this work were grown at 700 °C and a plasma power of 22 W, employing a gas flow ratio of 80 sccm C_2_H_2_ to 320 sccm NH_3_ at 5 mbar for 10 min.

### Cell culturing

MDA-MB-231 cells were seeded on MWCNTs-textured silicon scaffolds in a plastic Petri dish. In the above studies, the MWCNT-based surface constituted the scaffold under investigation (Group 1) and the Petri dish surface was the control experiment (Group 2). MDA-MB-231 cells were expanded for 1 week in a culture medium consisting of supplemented Dulbecco’s modified Eagle’s medium (DMEM). The medium was replaced every other day. MDA-MB-231 cells were plated on 13 mm-diameter coverslips at 30,000 cells/coverslip density. The cells were left for 1 h at 37 ^°^C to allow attachment after which 1 mL of pre-warmed culture medium was applied on each well. After 2 days, the cells were divided into two groups and cultured in the absence of MWCNTs-based scaffold (EXMWCNT control group) and in the presence of the MWCNTs-based scaffold (MWCNT group). To obtain a confluent monolayer, 2 × 10^5^ cells were plated onto the tissue culture dish and kept in an incubator at 37 °C and in an atmosphere which constituted 5% CO_2_, 95% air, and 100% humidity. The cells were incubated for 1 week and the culture medium was changed every other day. Each group contained six samples and the experiment was repeated three times.

### SEM analysis of cells cultured on a MWNCTs-based scaffold

After 1 week of culturing, the scaffolds were prepared as described previously [5]. Briefly, the scaffolds were washed once with PBS solution and fixed with 2.5% glutardialdehyde (75% solution in water, Sigma-Aldrich, Germany) buffered with 0.1 M cacodylate (Sigma-Aldrich, Germany), pH 7.4, for 2 h at 4 °C. The samples were then dehydrated by passing through a series of increasing concentration of ethanol (25, 50, 75, 95% each for 15 min). The analysis was performed on a LEO Zeiss Gemini 1530 scanning electron microscope (SEM).

### Immunocytochemistry

Immunocytochemistry was carried out with the streptavidin–biotin immunostaining protocol for the two groups (MWCNT and EXMWCNT), using MMP-2, MMP-9, PI3K, AKT and NF-κB primary antibodies. Each of the two group cells was fixed with 4% paraformaldehyde for 30 min. The samples were permeabilized by 0.1% Triton X-100 in Tris-buffered saline (TBS, 10 nM Tris–HCl, 150 nM NaCl, pH 7.4) for 10 min. Thereafter incubation with the secondary antibody was carried out overnight at 4 °C with blocking buffer (Life Technologies, USA) at room temperature for 1 h. Subsequently, the cells were stained with 5 μg/ml 3,3′-diaminobenzidine (DAB) for 2 min and images were recorded by a research microscope. The cell count of the MDA-MB-231 cells was performed according to immunostaining for MMP-2, MMP-9, PI3K, AKT and NF-κB. Expressions of these proteins on two groups (MWCNT and EXMWCNT) were evaluated semiquantitatively using H-score analysis. The immunostaining intensities were categorized by the following scores: 0 (no staining), 1 (weak, but detectable staining), 2 (moderate staining), and 3 (intense staining). An H-score value was derived for each specimen by calculating the sum of the percentage of cells for both MWCNT and EXMWCNT, which were stained at each intensity category, multiplied by their respective score, using the formula H-score = ∑*Pi* (*i* + 1), where ‘*i*’ indicates the intensity of staining value between 1, 2 or 3 corresponding to weak, moderate or strong staining, respectively. Pi is the percentage of stained cells for each intensity, varying from 0 to 100%. For each slide, five different fields were evaluated microscopically at 200× magnification. H-score evaluation was performed independently by at least two investigators (MO and AU) blinded to the source of the samples as well as to each other’s results; the average score of both was then used.

### Statistical analysis

Statistical analysis was performed using the Statistical Package for Social Science (SPSS for Windows, Version 15.0, Chicago, Illinois). The descriptive statistics for the normally distributed variables were expressed as the mean ± standard deviation. Kruskal–Wallis test was used for statistical comparisons of the groups where variables did not fit the normal distribution curve. Mann–Whitney *U* test with Bonferroni correction was used for post hoc tests. *P* value of < 0.05 is considered as statistically significant.

## Results

**Fig. 1:**
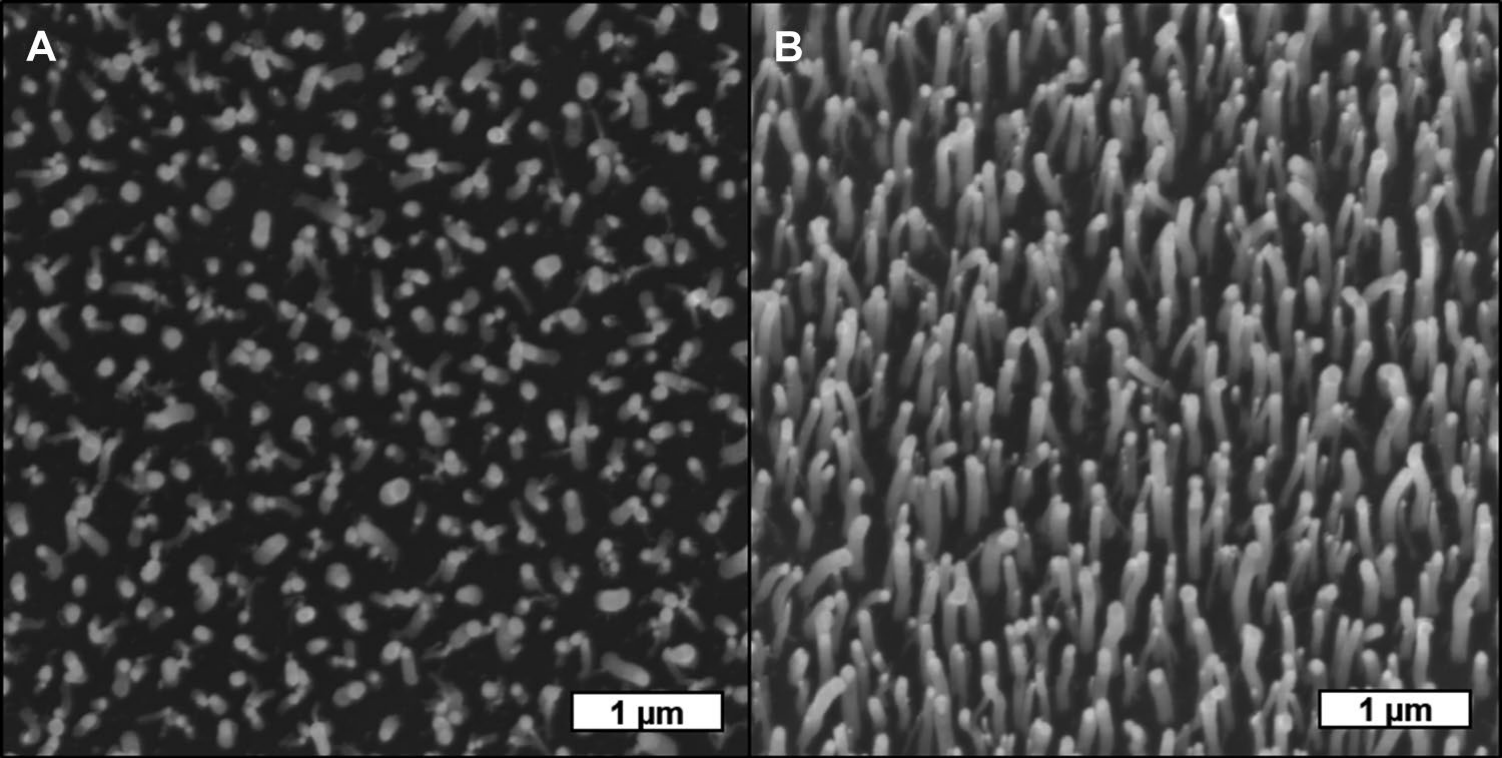
Scanning electron microscopy (SEM) images of vertically aligned MWCNTs of an approximate length of 2 µm and 80–200 nm diameter in **a** top and **b** 20° tilt perspective

**Fig. 2:**
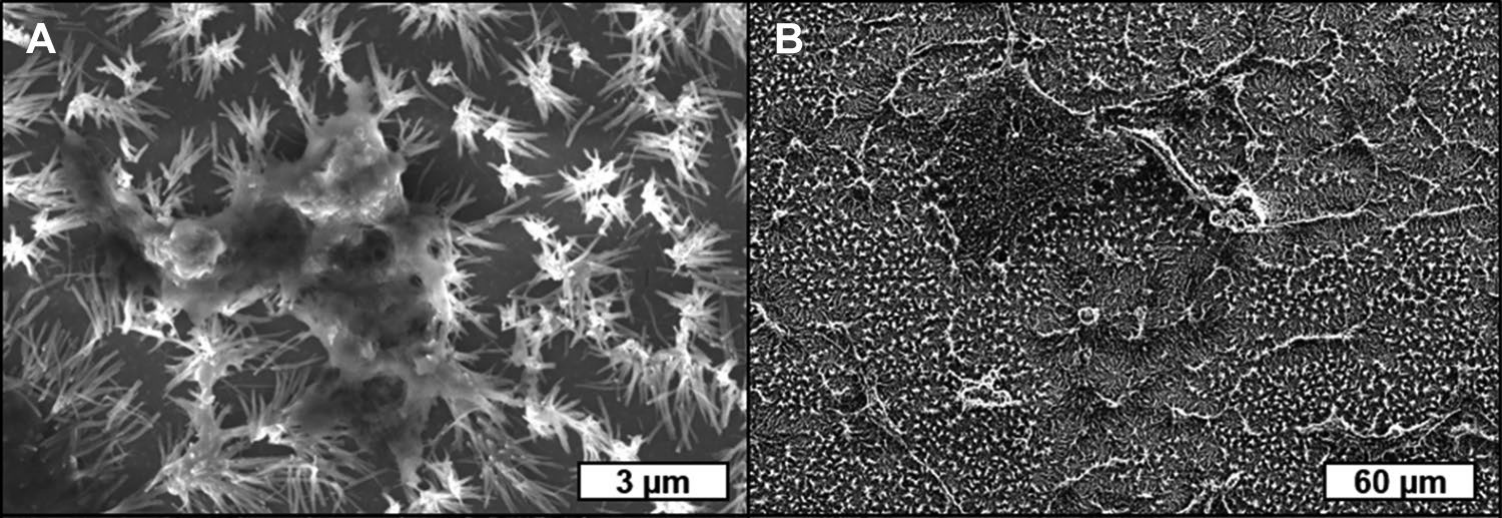
SEM images of MDA-MB-231 cells adhering to MWCNT-based scaffolds. It can be seen that the cells attach to the MWCNT bundles (**a**) and cover the surface of the scaffold homogenously (**b**)

The MWCNT scaffolds resemble a nanoscopic bed of nails where vertically aligned MWCNTs with 80–200 nm diameter and approximately 2 µm length would texture a plane of polished silicon surface. Scanning electron microscopy images can be seen in Fig. 1. MDA-MB-231 human breast cancer cells were incubated on the displayed MWCNT scaffolds. After incubation, the cells were fixed on both samples to enable SEM imaging. The corresponding SEM analysis in Fig. 2 reveals that the cells adhere on the MWCNT scaffold homogenously. It can be seen that the cells attach to the MWCNT-textured surface, growing along the surface while bending the MWCNTs toward them (Fig. 2).

**Fig. 3:**
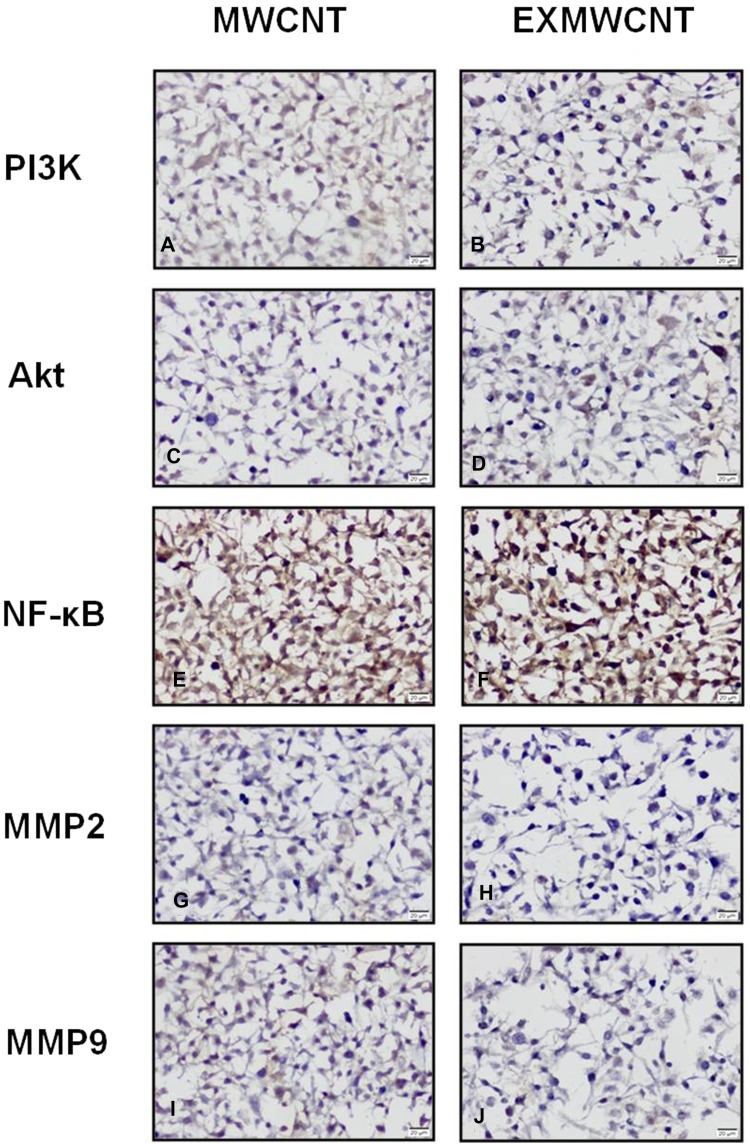
Immunohistochemical analysis of MDA-MB-231 cells adhering to multi-walled carbon nanotube-based scaffold (**a**, **c**, **e**, **g**, **i**) and in the absence of MWCNTs-based scaffold (**b**, **d**, **f**, **h**, **j**). Moderate PI3K (**a**, **b**), Akt (**c**, **d**), MMP2 (**g**, **h**) and MMP9 (**i**, **j**) expressions were seen in both MWCNTs and EXMWCNTs scaffolds. Strong NF-κB (**e**, **f**) immunoreactivity was seen by MDA-MB-231 cells on the MWCNTs and EXMWCNTs scaffolds (original magnification: ×200.)

**Fig. 4:**
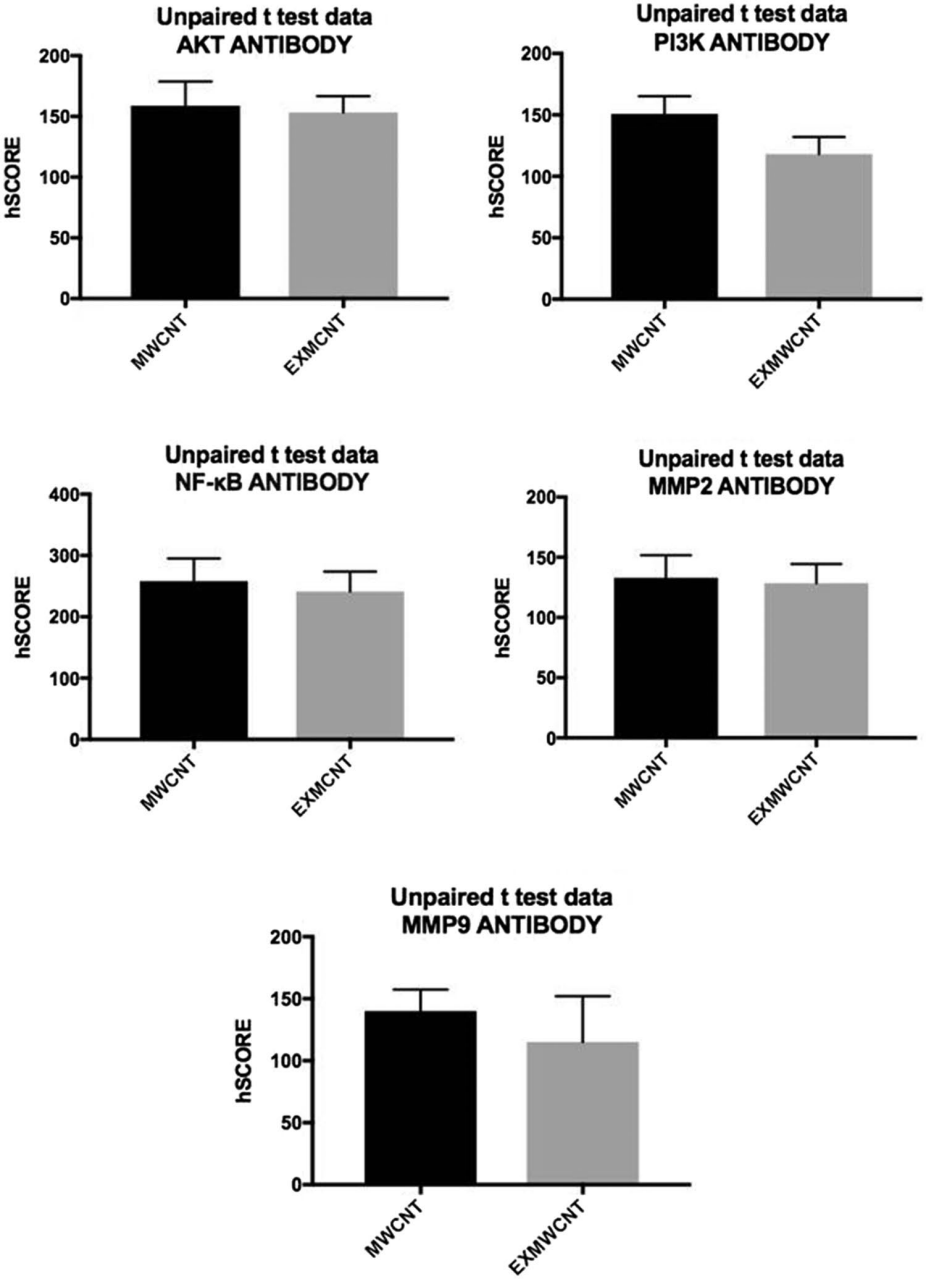
Comparison of H-score values of PI3K, AKT, NFKP, MMP2 and MMP9 primer antibodies in MDA-MB-231 cells on MWCNT and EXMWCNT cultures. There are not any differences between these groups except for the PI3K values

The immunofluorescence activity of the MDA-MB-231 cells on both supports have similar immunoreactivity staining with anti-AKT MMP-2, MMP-9 and NF-κB primary antibodies. These results did not show a significant difference between the two groups (Table 1 and Fig. 4). However, PI3K immunoreactivity in the presence of the MWCNTs-based scaffold was high among the MWCNTs-based scaffold group (Fig. 3a, b) (Table 1 and Fig. 4).

**Table 1:** H-score values of PI3K, Akt, NFKP, MMP2 and MMP9 primer antibodies in the MDA-MB-231 cells on MWCNT and EXMWCNT cultures

	MWCNT	EXMWCNT
PI3K	115.30 ± 36.658**	159.40 ± 30.475**
AKT	158.80 ± 19.887	153.20 ± 13.530
NFKB	258.20 ± 36.986	241.10 ± 32.726
MMP2	103.80 ± 35.418	116.10 ± 15.659
MMP9	140.10 ± 16.452	115.10 ± 35.086

**p < 0.001

## Discussion

The study of breast cancer metastasis is still difficult and has some limitations for in vitro and in vivo models. Therefore, we intended to develop a nanoscopic 3D MWCNT-based scaffold for breast cancer metastasis investigation. MDA-MB-231 cells were grown on the proposed MWCNT-based scaffold and the cells were characterized by immunohistochemical and scanning electron microscopic techniques. The aim of this study was to observe whether the MWCNT-based scaffold has a preferential structure for cell growth and provides a cytocompatible environment necessary for efficient human MDA-MB-231 cell lines.

An ideal model for breast cancer metastasis studies should be useful as a tool to study the entire metastasis process of cancer cells (Xia et al. 2012). Scanning electron microscopy images (Fig. 2) illustrate that MDA-MB-231 cells have invaded the proposed model scaffold which indicates that MWCNT scaffolds (Fig. 1) exhibit a good cell adhesion capability.

The metastatic MDA-MB-231 cells grew and migrated quickly throughout the entire scaffold. In addition, these cells also proliferated, migrated and infiltrated the culture dish (EXMWCNT group). The proposed MWCNT scaffolds can significantly distinguish the invasion and the growth difference of MDA-MB-231 cells, which would be very promising for BrCa cell metastasis study.

MDA-MB-231 cell properties on MWCNT-based scaffolds were investigated for proliferation, migration and invasion of the cells using immunohistochemical techniques. First, it is observed that MDA-MB-231 cells have high AKT expression on MWCNT scaffolds with no difference compared to the EXMWCNT group. AKT seems to play an important role in the migration and invasion of breast cancer leading to metastasis (Wang et al. 2014). It is known that AKT can suppress the elevated rate of apoptotic cell death, whereas AKT does not result in the induction of mammary tumors (Hutchinson et al. 2001). Moreover, high levels of AKT seem to show a resistance toward many different types of breast cancer treatment methods including tamoxifen (deGraffenried et al. 2004) [19].

Secondly, in this study, the PI3K immunoreactivity in MDA-MB-231 cells on both MWCNT and EXMWCNT scaffolds was investigated and it was found that the immunoreactivity was significantly different in the MWCNT group compared to the EXMWCNT group. It is known that the PI3K/AKT/(mTOR) pathway is aberrantly activated in numerous human cancers, including breast cancer (Adeyinka et al. 2002). This pathway plays a central role in diverse cellular functions including proliferation, growth, survival and metabolism, where it shows a central part in signal transduction in the cellular metabolism (Won et al. 2012). The PI3K–AKT pathway provides good therapeutic responses in clinical trials (Britten 2013), and the activation of PI3K–AKT pathway has a critical role in providing an alternative survival pathway in cancer cells (Hoeflich et al. 2009). Our PI3K immunoreactivity results claim that the MWCNT-based scaffold induces the PI3K immunoreactivity for MDA-MB-231 cells, where these MWCNTs can be used in cancer studies.

Furthermore, the transcription factor NF-κB plays an important role in the innate immunity and is a key regulator for inflammation, which is highly expressed in many breast cancer types promoting cell invasion and metastasis (Khan et al. 2009). Activated NF-κB binds to the promoter region of downstream genes and initiates the transcription of target genes responsible for inflammation, immunoregulation, cell differentiation and migration (Meylan et al. 2009). Li at al. (2014) have reported that NF-κB is hyperactivated by upregulating Notch-1 in human breast cancer cells MDA-MB-231, leading to robust cell proliferation, invasion and adhesion. It is known that NF-κB inactivation inhibits cell invasion completely, and activation of NF-κB stimulates the expression of MMP-2 to facilitate cell invasion. In this context, it has been revealed that NF-κB activation in MDA-MB-231 cell lines does not differ from that of the MWCNT and EXMWCNT scaffolds.

Also, MMP-2/9 has an important extracellular matrix homeostatic function during muscle growth, development and repair processes. It also plays an important role in the processes of invasive metastasis and angiogenesis in various tumors (Deryugina and Quigley 2006). Activated MMP-2/9 facilitates the passage of tumor cells through the ECM and the basal membrane of the blood vessel wall, promoting the invasion of tumor cells. Specifically, MMP-2 may act directly by degrading ECM components like type III collagen or indirectly by activating pro-MMP-2 or by inducing tumor angiogenesis through vascular endothelial growth factors (Tetu et al. 2006). Additionally, overexpression of MMP-2 has been correlated with a higher probability of having N2–3 or stage III (Mohammad et al. 2012). Our observations indicate that MMP-2/9 expressions of MDA-MB-231 cells are increased in the MWCNT group as compared to the EXMWCNT group, but the values are not considered to be statistically significant. In this context, recently Zhang et al. have reported that MMP-2/-9 overexpression and cell invasiveness can be detected in di(2-ethylhexyl)phthalate (which are chemically estrogenic treated cells), suggesting that the MMP-2/-9 overexpression leads to enhanced invasion of MDA-MB-231 cells (Zhang et al.^2016^).

Ultimately, the hyperactivation of the PI3K/AKT pathway stimulates the transactivation potential of NF-κB which initiates the expression of MMP-2/9 to facilitate cell invasion.

## Conclusion

In this paper, the 3D nanoscale MWCNT-based scaffold for tumor progression of MDA-MB-231 breast cancer cells was investigated. Scanning electron microscopy showed that the cells adhered homogeneously onto MWCNT-textured surfaces. With immunohistochemical techniques, the molecular differences for cells grown on scaffolds, with and without MWCNTs, were observed and MWCNT-based scaffolds induced PI3K immunoreactivity. However, no significant difference was found between AKT, NF-κB, MMP2 and MMP9. Therefore, it is claimed that MDA-MB-231 cells and the molecular characteristics of these cells are not affected by a nano 3D MWCNT-based scaffold. In conclusion, the MWCNT-based scaffolds enhance the cell adhesion onto the scaffold and exhibit more biomimetic and physiologically relevant features which may be used for in vitro BrCa cell metastatic studies.

## References

[CR1] Adeyinka A, Nui Y, Cherlet T, Snell L, Watson PH, Murphy LC (2002). Activated mitogen-activated protein kinase expression during human breast tumorigenesis and breast cancer progression. Clin Cancer Res.

[CR2] Baselga J (2011). Targeting the phosphoinositide-3 (PI3) kinase pathway in breast cancer. Oncologist.

[CR3] Britten CD (2013). PI3K, MEK inhibitor combinations: examining the evidence in selected tumor types. Cancer Chemother Pharmacol.

[CR4] Cizkova M, Cizeron-Clairac G, Vacher S, Susini A, Andrieu C, Lidereau R (2010). Gene expression profiling reveals new aspects of PIK3CA mutation in ERalpha-positive breast cancer: major implication of the Wnt signaling pathway. PLoS One.

[CR5] Correa-Duarte MA, Wagner N, Rojas-Chapana J, Morsczeck C, Thie M, Giersig M (2004). Fabrication and biocompatibility of carbon nanotube-based 3D networks as scaffolds for cell seeding and growth. Nano Lett.

[CR6] deGraffenried LA, Friedrichs WE, Russell DH, Donzis EJ, Middleton AK, Silva JM (2004). Inhibition of mTOR activity restores tamoxifen response in breast cancer cells with aberrant Akt Activity. Clin Cancer Res.

[CR7] Deryugina EI, Quigley JP (2006). Matrix metalloproteinases and tumor metastasis. Cancer Metastasis Rev.

[CR8] Firkowska I, Olek M, Pazos-Perez N, Rojas-Chapana J, Giersig M (2006). Highly ordered MWNT-based matrixes: topography at the nanoscale conceived for tissue engineering. Langmuir.

[CR9] Firkowska I, Giannona S, Rojas-Chapana J, Luecke K, Brüstle O, Giersig M, Giersig M, Khomutov GB (2008). Biocompatible nanomaterials and nanodevices promising for biomedical applications. Nanomaterials for application in medicine and biology.

[CR10] Hoeflich KP, O’Brien C, Boyd Z, Cavet G, Guerrero S, Jung K (2009). In vivo antitumor activity of MEK and phosphatidylinositol 3-kinase inhibitors in basal-like breast cancer models. Clin Cancer Res.

[CR11] Hoesel B, Schmid JA (2013). The complexity of NF-kappa B signaling in inflammation and cancer. Mol Cancer.

[CR12] Hutchinson J, Jin J, Cardiff RD, Woodgett JR, Muller WJ (2001). Activation of Akt (protein kinase B) in mammary epithelium provides a critical cell survival signal required for tumor progression. Mol Cell Biol.

[CR13] Khan GN, Gorin MA, Rosenthal D, Pan Q, Bao LW, Wu ZF (2009). Pomegranate fruit extract impairs invasion and motility in human breast cancer. Integr Cancer Ther.

[CR14] Khanna C, Hunter K (2005). Modeling metastasis in vivo. Carcinogenesis.

[CR15] Li HC, Cao DC, Liu Y, Hou YF, Wu J, Lu JS (2004). Prognostic value of matrix metalloproteinases (MMP-2 and MMP-9) in patients with lymph node-negative breast carcinoma. Breast Cancer Res Treat.

[CR16] Li L, Zhao F, Lu J, Li T, Yang H, Wu C (2014). Notch-1 signaling promotes the malignant features of human breast cancer through NF-jB activation. PLoS One.

[CR17] Lipton A, Uzzo R, Amato RJ, Ellis GK, Hakimian B, Roodman GD (2009). The science and practice of bone health in oncology: managing bone loss and metastasis in patients with solid tumors. J Natl Compr Canc Netw.

[CR18] Meylan E, Dooley AL, Feldser DM, Shen L, Turk E, Ouyang C (2009). Requirement for NF-jB signalling in a mouse model of lung adenocarcinoma. Nature.

[CR19] Mohammad MA, Zeeneldin AA, Abd Elmageed ZY, Khalil EH, Mahdy SM, Sharada HM (2012). Clinical relevance of cyclooxygenase-2 and matrix metalloproteinases (MMP-2 and MT1-MMP) in human breast cancer tissue. Mol Cell Biochem.

[CR20] Olek M, Ostrander J, Jurga S, Moehwald H, Kotov N, Kempa K (2004). Layer-by-layer assembled composites from multiwall carbon nanotubes with different morphologies. Nano Lett.

[CR21] Pavoor P, Gearing BP, Gorga RE, Bellare A, Cohen REJ (2004). Engineering the friction-and-wear behavior of polyelectrolyte multilayer nanoassemblies through block copolymer surface capping, metallic nanoparticles, and multiwall carbon nanotubes. Appl Polym Sci.

[CR22] Shin SY, Kim CG, Jung YJ, Lim Y, Lee YH (2016). The UPR inducer DPP23 inhibits the metastatic potential of MDA-MB-231 human breast cancer cells by targeting the Akt-IKK-NF-κB-MMP-9 axis. Sci Rep.

[CR23] Tetu B, Brisson J, Wang CS, Lapointe H, Beaudry G, Blanchette C (2006). The influence of MMP-14, TIMP-2 and MMP-2 expression on breast cancer prognosis. Breast Cancer Res.

[CR24] Torre LA, Bray F, Siegel RL, Ferlay J, Lortet-Tieulent J, Jemal A (2015). Global cancer statistics, 2012. CA Cancer J Clin.

[CR25] Trzeciak T, Rybka JD, Akinoglu EM, Richter M, Kaczmarczyk J, Giersig M (2016). In vitro evaluation of carbon nanotube-based scaffolds for cartilage tissue engineering. J Nanosci Nanotechnol.

[CR26] Wang M, Cheng X, Zhu W, Holmes B, Keidar M, Zhang LG (2014). Design of biomimetic and bioactive cold plasma-modified nanostructured scaffolds for enhanced osteogenic differentiation of bone marrow-derived mesenchymal stem cells. Tissue Eng Part A.

[CR27] Won JK, Yang HW, Shin SY, Lee JH, Heo WD, Cho KH (2012). The cross-regulation between ERK, PI3K signaling pathways determines the tumoricidal efficacy of MEK inhibitor. J Mol Cell Biol.

[CR28] Xia TS, Wang GZ, Ding Q, Liu XA, Zhou WB, Zhang YF (2012). Bone metastasis in a novel breast cancer mouse model containing human breast and human bone. Breast Cancer Res Treat.

[CR29] Xie Y, Naizabekov S, Chen Z, Tokay T (2016). Power of PTEN/AKT: molecular switch between tumor suppressors and oncogenes. Oncol Lett.

[CR30] Zhang S, Ma J, Fu Z, Zhang Z, Cao J, Huang L (2016). Promotion of breast cancer cells MDA-MB-231 invasion by di (2-ethylhexyl)phthalate through matrix metallo-proteinase-2/-9 overexpression. Environ Sci Pollut Res Int.

